# Characterization of ultrasound and postnatal pathology in fetuses with heterotaxy syndrome

**DOI:** 10.3389/fcvm.2023.1195191

**Published:** 2023-06-26

**Authors:** Qiumei Wu, Shan Guo, Biying Huang, Wen Ling, Longzhuang Peng, Hong Ma, Fa Chen, Guorong Lyu, Min Liu, Xiuqing Qiu, Zongjie Weng

**Affiliations:** ^1^ Department of Medical Ultrasonics, Fujian Maternity and Child Health Hospital, College of Clinical Medicine for Obstetrics & Gynecology and Pediatrics, Fujian Medical University, Fuzhou, China; ^2^ Department of Pathology, Fujian Maternity and Child Health Hospital, College of Clinical Medicine for Obstetrics & Gynecology and Pediatrics, Fujian Medical University, Fuzhou, China; ^3^ Department of Epidemiology and Health Statistics, School of Public Health, Fujian Medical University, Fuzhou, China; ^4^ Department of Medical Ultrasonics, The Second Affiliated Hospital of Fujian Medical University, Quanzhou, China; ^5^ Department of Clinical Medicine, Quanzhou Medical College, Quanzhou, China; ^6^ Department of Obstetrics & Gynecology, Fujian Maternity and Child Health Hospital, College of Clinical Medicine for Obstetrics & Gynecology and Pediatrics, Fujian Medical University, Fuzhou, China

**Keywords:** heterotaxy syndrome, anatomic casting, five-step ultrasound screening, left isomerism, right isomerism

## Abstract

**Background:**

To explore the diagnostic clues and abnormality spectrum of heterotaxy syndrome by prenatal ultrasonography and postnatal verification.

**Methods:**

The prenatal ultrasonic data of 88 heterotaxy syndrome fetuses were analyzed retrospectively as left isomerism (LI) and right isomerism (RI). Prenatal ultrasound compared with the anatomical casting of the fetal body after labor induction, and the confirmatory postnatal diagnosis after delivery.

**Results:**

Fetal LI showed typical malformations of gastric vesicles on different sides from the heart, absence of hepatic segment of the inferior vena cava (IVC), abdominal aorta (AO) parallel with the azygos vein (AV), bilateral left bronchus, bilateral left atrial appendages, and polysplenia; intracardiac malformations of AV septal defects (AVSD), single atrium (SA), left ventricular outflow tract obstruction (LVOTO), and double-outlet right ventricle (DORV); and cardiac conduction abnormalities of sinus bradycardia and AV blockage. Fetal RI reported typical malformations of gastric vesicles on different sides from the heart, juxtaposition of the IVC with AO, anomalous pulmonary venous connection (APVC), asplenia, and bilateral right atrial appendages; intracardiac malformations of AVSD, SA, single ventricle, pulmonary atresia and stenosis, and DORV. The postnatal verification revealed 3 malformations misdiagnoses and 4 malformations missed diagnoses in LI fetuses and 10 misdiagnoses and 8 missed diagnoses in RI fetuses.

**Conclusions:**

The proposed five-step prenatal ultrasonography has an important diagnostic value for the identification and classification of heterotaxy syndrome. The different sides of gastric vesicles and cardiac apex are important diagnostic clues for heterotaxy syndrome, featuring disconnected or hypoplastic IVC, typical complex cardiac malformation, and atrioventricular block in fetal LI, and shown APVC, juxtaposition of IVC and AO, and intracardiac malformations such as AVSD, DORV, and LVOTO in fetal RI.

## Introduction

Heterotaxy syndrome (HS) is a rare and complex condition with abnormal arrangement of organs in relation to the left-right axis of the body, different from complete situs solitus or situs inversus which occurs 0.8 in 10,000 at live birth (1). HS has two recognized types: left isomerism (LI) and right isomerism (RI). The severity of the cardiac malformation is the main determinant of the outcomes (2). Overall, the five-year survival rate of RI and LI fetuses ranges within 30%–74% and 65%–84%, respectively (3, 4). As the treatment regimens for and prognoses of LI and RI fetuses vary greatly (5, 6), an accurate identification and classification of HS cases warrant due attention. LI and RI involve a wide range of malformations, as well as an array of overlapping disease features. Some cases have atypical characteristics, making a clear distinction between left and right isomers even more difficult (7, 8). Studies have proposed distinguishing HS into LI or RI by evaluating the morphology of atrial appendages (9, 10). In addition, another study further proposed classification by assessing the shape of the atrial appendages and the venous atrial connection (11). However, due to the small size, a location beyond the standard planes, and the not-always-coordinated arrangement of organs with the atrial appendages, atrial appendages cannot be reliably identified by prenatal ultrasonography, posing considerable challenges to accurate identification and classification of HS (12).

A combination of the cardiac sequential segmental method with the extracardiac characteristic sonography has been explored in HS fetuses (13, 14), in which an LI diagnosis was indicated in the presence of at least two of the following features: (1) complete atrioventricular septal defect or other structural heart diseases; (2) interrupted inferior vena cava (IVC) with azygos continuation; (3) early fetal heart block; (4) viscerocardiac heterotaxy, while a RI was suspected in the presence of at least two of the following: (1) complete atrioventricular septal defect or other structural heart diseases; (2) juxtaposition of inferior vena cava and descending aorta; (3) viscerocardiac heterotaxy. Studies have found atrioventricular heart block and IVC interruption to be typical characters of LI, and complete anomalous pulmonary venous connection and juxtaposition of inferior vena cava and aorta to be typical signs of RI, which also could be consistent by varying imaging approach (12, 15–17). However, due to the diversity in the arrangement and combination of multiple malformations in HS fetuses, the general characteristics of classical HS may not be manifested in more than 20% of HS patients (18), signifying that these existing classification methods do not apply to all HS fetuses (19). Given the malformation diversities and challenges in the identification and classification of HS, the aim of this approach to propose a comprehensive and sequential five-step prenatal ultrasonic method to diagnose and classify fetuses with suspected HS, which delineates the disease characteristics of each organ system and depicts each malformation independently. The proposed method can provide a reliable basis for prenatal consultation and clinical decision-making.

## Methods

This retrospective study analyzed all HS fetuses diagnosed by the prenatal five-step approach using ultrasonography in Fujian Maternity and Child Health Hospital, Fujian Medical University between January 2014 and May 2022. Informed consent forms were obtained from the guardians of all study subjects. The Ethics Committee of Fujian Maternity and Child Health Hospital, Fujian Medical University (2014FY110700) approved the study. Data on prenatal diagnosis and postnatal follow-ups were obtained from the electronic medical record. Postnatal validation methods include anatomical casting and Ultrasonography. HS patients were excluded from the study if the data from antenatal or postnatal follow-ups were unavailable.

### Prenatal ultrasonography

All patients underwent ultrasound screening performed with GE Voluson E8 or E10, Siemens ACUSON S2000, Philips IE33 ultrasound machine with a 4–8 MHz transabdominal probe. Ultrasound settings for fetal echocardiography and pregnancy examination were set according to the parameters recommended by the manufacturer. Prenatal Ultrasound was performed according to the AIUM Performance of Fetal Echocardiography guideline (20), which was performed using the segmental analysis method, combined with Color Doppler and Power Doppler. The atrial and ventricular movements were recorded in M-mode to observe the cardiac rhythm (21). The diagnostic criteria proposed by Berg et al. were adopted for diagnosing HS (12).

Five step approach by Echocardiography: Step 1: The position of viscera and heart was examined to determine: (1) the position of abdominal organs, the course of the portal vein (PV), and the development of IVC and its position in relation to the abdominal aorta; (2) the position of the heart. Step 2: The systemic and pulmonary venous return was examined to detect: (1) the structure of IVC or the interrupted IVC with continuity of the azygos vein (AV). The latter was manifested as a “double vessel” sign in the chest and drainage of the umbilical vein to the right atrium in the sagittal view of the fetal abdomen on a color Doppler flow diagram, showing an absence of the upper part of the inferior vena cava; (2) the drainage characteristics of venous ducts (VD) and hepatic vein (HV) and their connection with atrium; (3) the location and number of superior vena cava and its connection with atrium; (4) the connection between the pulmonary vein and the atrium or systemic vein, and the centrifugal venous flow in the mediastinum or the hepatic inflow in the liver except the umbilical vein and portal vein. Step 3: Signs of any intracardiac malformations were examined for: (1) a common atrium; (2) a single ventricle, two symmetrical ventricles, or a ventricular septal defect (its size and location, if indicated); (3) regurgitation in AV valve or a common AV valve in determining the type of AV valve and its connection with the ventricle; (4) blockage of the outflow tract; (5) defects in aortic and pulmonary artery trunks and branches; (6) displacement of aortic arch; (7) abnormalities in the position and blood flow direction of the ductus arteriosus. Step 4: The fetal heart rate and heart rhythm were measured. In case of abnormality, M-mode ultrasonography was performed to determine the type of arrhythmia. Step 5: Other abnormal signs were surveyed in the spleen, auricle, bronchus, gallbladder, and other organs. Postnatal verification.

### Pathological anatomy and vascular casting

The pathological anatomy of the specimens was combined with in-situ observation and post-mortem examination after *in vitro* fixation. The main steps of the vascular casting were as follows: (1) the umbilical vein was separated, into which a tube was inserted, ligated, and fixed with a silk thread. Afterwards, normal saline was injected into the tube to rinse the blood vessels until the color of the heart and lungs turned lighter; (2) the rinsed samples were cast with epoxy resin, polyamide resin curing agent, and ethyl acetate as the main hardening materials, and red pigment as casting agent; (3) the thoracic and abdominal organs were subsequently removed and corroded in potassium hydroxide solution. The features of intracardiac malformation and systemic and pulmonary venous connection routes were observed after the molding. The samples were photographed and archived before and after casting, and the casting results were recorded. The pathological anatomic diagnosis was referred to the published standards (22). Postnatal imaging data: In case of refusal of anatomical casting after induction of labour, the specimens were examined by conventional thoracic and abdominal ultrasonography. A routine physical examination was performed for live newborns, and an imaging examination was selected according to specific conditions, including abdominal ultrasonography, echocardiography, and electrocardiography.

### Statistical analysis

All statistical analysis was performed using SPSS v25.0 software. Comparisons were performed using Student *t*-tests assuming unequal variance between groups and Fisher’s exact chi-square. Quantitative data were expressed as mean ± standard deviation, and inter-group comparisons were analyzed by t-test. *P* < 0.05 was considered statistically significant.

## Results

**Figure 1:**
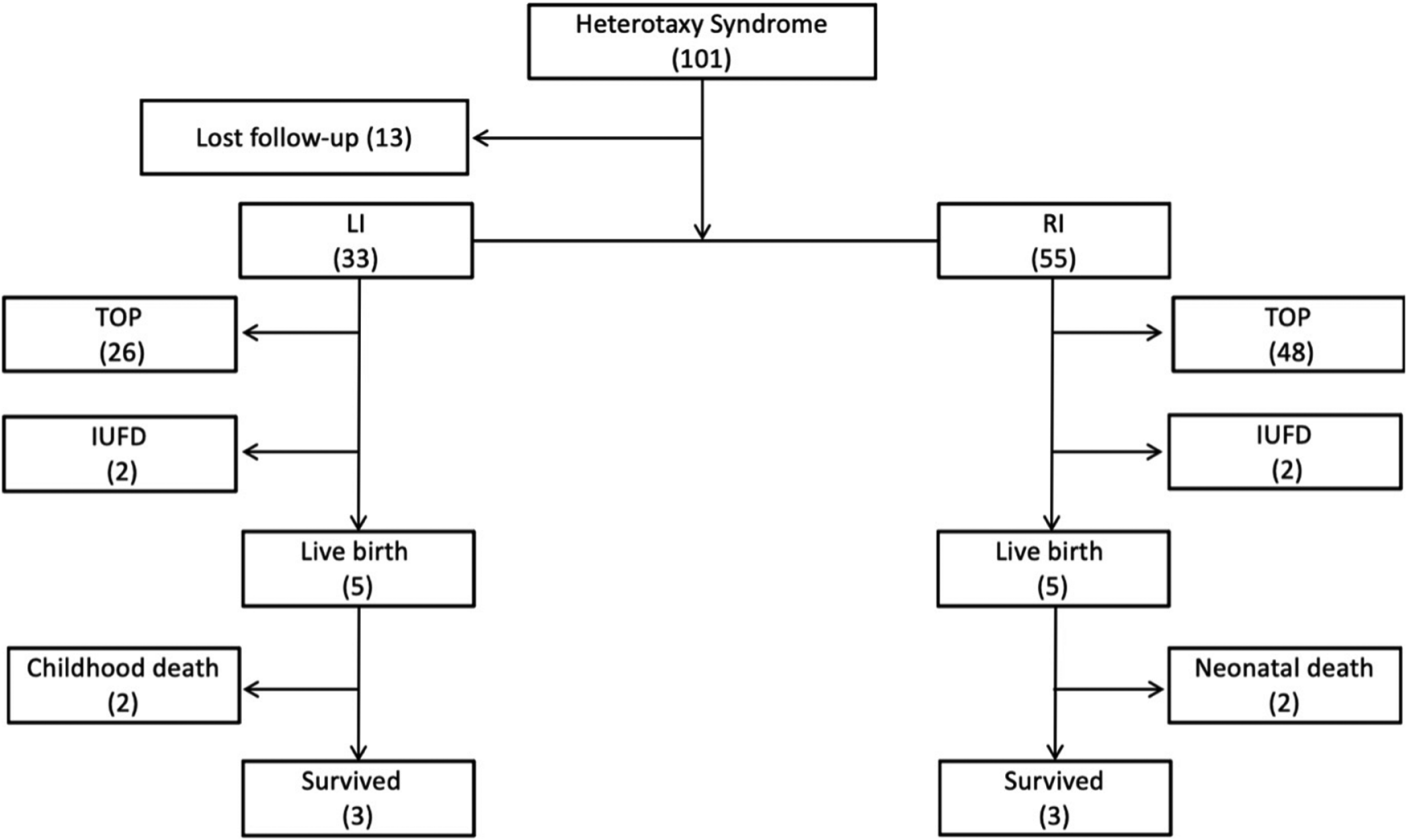
Flowchart of patients with the prenatal diagnosis of heterotaxy syndrome in this cohort. LI, left isomerism; RI, right isomerism; TOP, termination of pregnancy; IUFD, intrauterine fetal demise.

The flowchart of this study is shown in Figure 1. Thirteen fetuses were excluded from further analysis due to a lack of follow-up data. A total of 88 fetuses (33 LI, 37.5%; 55 RI, 62.5%) were included. Of the 33 LI cases, there were two intrauterine fetal demises (IUFDs) due to atrioventricular blockage, 26 pregnancy terminations and five live births. Among the five live births, there were two cases of normal cardiac formations, one case of the ventricular septal defect, and one double superior vena cava, who grew up well until the present study. The remanent case died at over one-year-old due to repeated postoperative cholangitis and liver failure, whose diagnosis included heterotaxy, intestinal malrotation, duodenal portal vein, omphalocele, and type I biliary atresia. Of the 55 RI cases, there were two IUFDs, 48 pregnancy terminations, and five live births with complex cardiac malformation. Two died in the newborn period, one was treated conservatively after birth, and the other underwent Glenn operation after birth. The latter three cases reported acceptable general health conditions up to the study. The mean gestational age at diagnosis of LI and RI fetuses was 24.56 ± 6.7 weeks (range: 15–34 weeks) and 23.03 ± 3.15 weeks (range: 13–28 weeks), respectively (*P* = 0.14). There were 4 cases of gender unknown in LI, and 5 cases in RI. The proportion of males and females in LI cases was similar (14/29 vs. 15/29, *p* > 0.05), while the number of males was far more than females in RI cases (33/50 vs. 17/50, *p* = 0.003).

### Prenatal findings of fetal left isomerism

**Figure 2:**
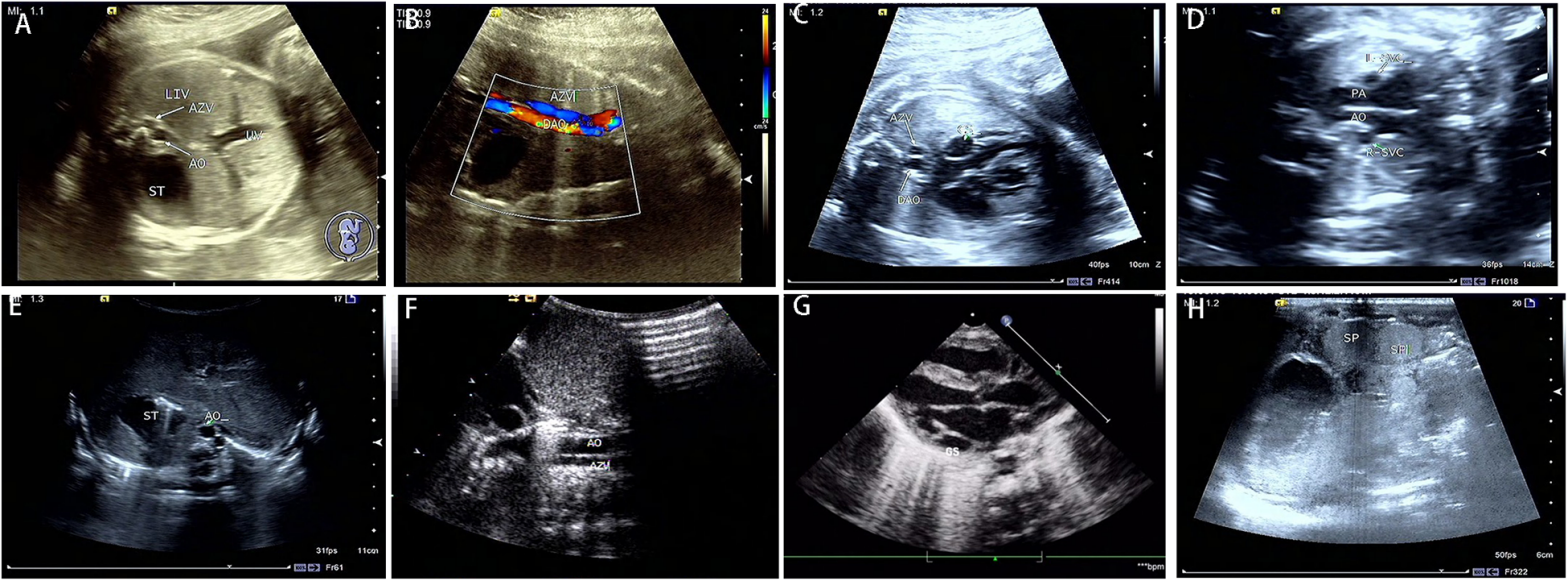
Prenatal and postnatal ultrasonography of LI fetuses. Prenatal sonography in the transverse section of the abdomen (**A**–**D**). (**A**) absence of a hepatic segment of IVC with dilated azygos; ST gastric vesicle, AO, abdominal aorta, AV azygos vein, and UV umbilical vein. (**B**) the parallel arrangement of the azygos vein and descending aorta. DAO, descending aorta. (**C**) the dilatation of coronary sinus. CS, coronary sinus. (**D**) double superior vena cava, L-SVC, left superior vena cava; R-SVC, right superior vena cava; PA, pulmonary artery. Postpartum sonograms (**E**–**H**): the images by postpartum sonography were consistent with prenatal ones.

The spectrum of cardiovascular and extracardiac lesions screened prenatally. Typical extracardiac malformations by ultrasonography in the 33 LI fetuses were as follows: gastric vesicles on different sides from the heart, absence of hepatic segment of the IVC, a parallel arrangement of the abdominal AO and the (semi) AV (Figures 2A,B), and double superior vena cava (Figure 2D). Further exploration of the venous drainage route in these LI fetuses revealed: hepatic veins infused separately with both atriums (6%); hepatic veins and ductus venous (DV) were separately introduced into both atriums (3%); or bilateral pulmonary veins flowed separately into both atriums (12%). Typical intracardiac malformations included atrioventricular septal defect, single atrium, left ventricular outflow tract obstruction, pulmonary atresia, double-outlet right ventricle, and bilateral left atrial appendage. Cardiac conduction abnormalities in the LI fetuses included sinus bradycardia and atrioventricular blockage. Other systemic abnormalities involved typical bilateral left bronchi, midline liver, and polysplenia. Atypical ultrasonographical signs detected included hypoplasia of the hepatic segment of the inferior vena cava with AV dilation, normal spleen morphology, and nondetectable atria and bronchi.

### Anatomy and casting characterization of fetal left isomerism

**Figure 3:**
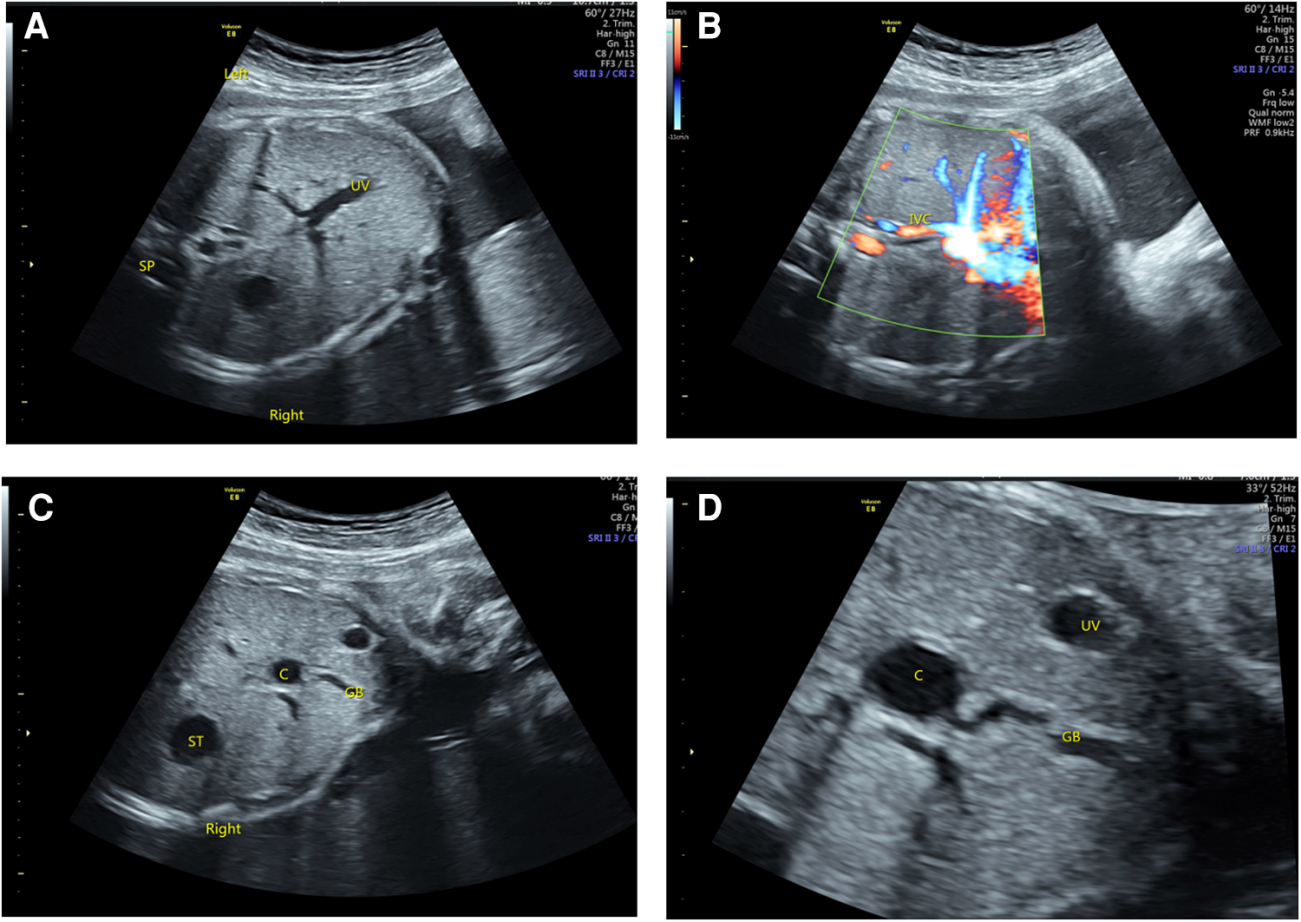
Ultrasonography of BASM in LAI fetuses. (**A**) The right-sided stomach and the portal vein branch show a “T” sign. (**B**) The IVC was small and flowed back to the right atrium after collecting hepatic vein blood. (**C**,**D**) A cystic structure was observed in the liver hilum, which was related to the gallbladder.

Of the 26 terminated cases, 20 cases received autopsy (76.92%), and the remaining six cases underwent vascular casting (23.08%), which confirmed the prenatal diagnosis (100%). The typical malformations of LI included inconsistent positions of gastric vesicles and the heart, bilateral bi-lobar lungs, bilateral left atrial appendages, absence of a hepatic segment of IVC with dilatation of azygos or semi-azygos veins, intracardiac abnormalities (atrioventricular septal defect with left ventricular outflow tract obstruction), and extracardiac malformations (midline liver, polysplenia, intestinal malrotation, short pancreas, etc.). Postnatal pathological anatomy reported atypical left morphological structure in some cases: two cases (8%) of hypoplasia of the hepatic segment of the IVC turned out to be thin and narrow; three cases (12%) of small left atrial appendage and unclear morphological features did not feature bilateral left atrial appendage morphology; 10 cases (38%) of polysplenia at the back of stomach turned out basically normal; three cases (12%) of bilateral lobar insufficiency were not the typical bilateral bi-lobar lung. Two intracardiac malformations were misdiagnosed: one case of the single ventricle was mistaken as an unbalanced atrioventricular septal defect and one case of pulmonary atresia as pulmonary artery stenosis; four intracardiac malformations were overlooked, including one case of anomalous pulmonary venous connection and three cases of abnormal hepatic venous return. Among the extracardiac malformations, one case of type I biliary atresia was misdiagnosed as a choledochal cyst (Figure 3).

### Prenatal ultrasound findings of fetal right isomerism

**Figure 4:**
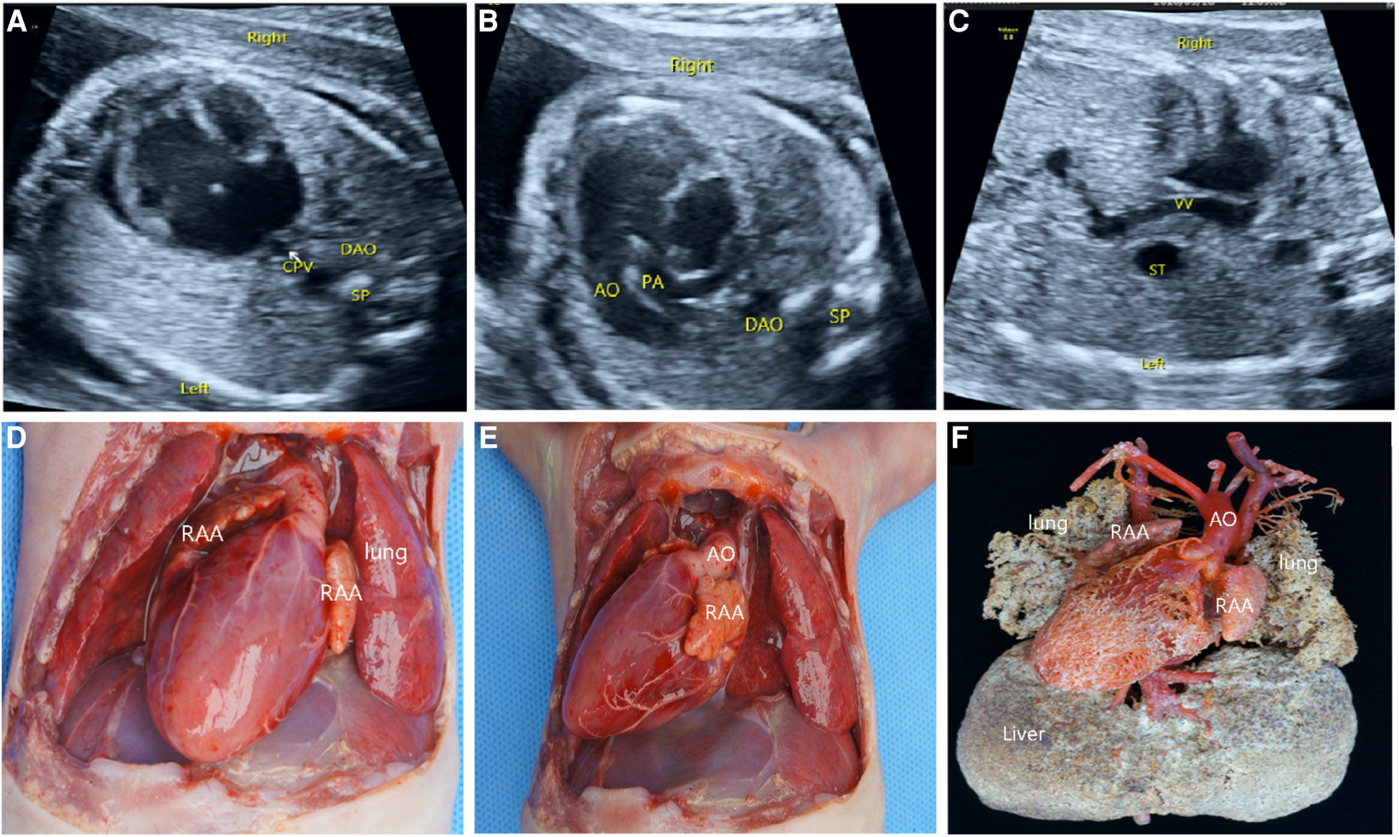
Prenatal ultrasonography and anatomical and vascular casting images of RI fetuses. (**A**) the “+” cross in the center of the heart disappeared during diastole in the four-chamber view, which was a complete endocardial cushion defect. (**B**) Common pulmonary vein entered the portal vein downwards via the descending vertical vein. (**C**) AO and pulmonary artery from the right ventricle. Figure (**D**–**F**): Front view of the specimen: right-sided heart, midline liver, bilateral right atrial appendage, aorta emanating from the front of the ventricle, left aortic arch, pulmonary artery at the rear, and double superior vena cava were seen. The back view of the specimen: four pulmonary veins formed a common pulmonary vein and flowed downwards via the descending vertical vein to the portal vein. CPV, a common pulmonary vein; VV, descending vertical vein.

As shown in Table 1, the typical signs of extracardiac malformations in RI fetuses included stomach bubbles on a different side from the heart, the juxtaposition of IVC and descending aorta, and bilateral superior vena cava (Figure 4A). The exploration of venous drainage reported 32 cases (58%) of hepatic venous connection to both atriums and 27 cases (82%) of anomalous pulmonary venous connection (APVC), including 10 cases (18%) of mixed APVC, 7 cases (13%) of complete superior cardiac APVC that drained to superior vena cava, and 10 cases (18%) of complete subcardiac APVC that finally converged into the portal vein (Figure 4B). Typical intracardiac malformations in the RI fetuses were as follows: atrioventricular septal defects, single atrium, single ventricle, pulmonary atresia and pulmonary stenosis (Figure 4C), and double-outlet right ventricular. No arrhythmia was found. In addition, bilateral right atrial appendages, midline liver, asplenia, and bilateral right bronchi were also reported. Atypical ultrasound anomalies of the RI fetuses were common pulmonary venous atresia, normal spleen, undetermined atria and bronchial morphology.

**Table 1:** Prenatal ultrasound characterization of left and right isomerism.

	LI	RI	*P* value
The position of gastric vesicles and heart			
Inconsistent			
Levocardia and right-side gastric vesicles	12/33	22/55	0.82
Mesocardiac and right-side gastric vesicles	2/33	10/55	0.20
Dextrocardia and left-side gastric vesicles	6/33	12/55	0.79
Consistent			
Levocardia	10/33	9/55	0.18
Dextrocardia	3/33	3/55	0.67
Midline liver	28/33	35/55	0.0497
Juxtaposition AO /IVC	0/33	48/55	<0.0001
IVC			
Interrupted IVC	31/33	0/55	<0.0001
Hypoplasia of IVC	2/33	0/55	0.138
SVC			
Single left SVC	5/33	9/55	>0.9999
Single right SVC	17/33	18/55	0.115
Bilateral SVC	11/33	28/55	0.11
Hepatic veins			
HV and DV directly flowing into one atrium	30/33	13/55	<0.0001
HV and DV flowing into both atriums or one atrium	3/33	42/55	<0.0001
Pulmonary veins			
Bilateral PVs flowing into one atrium	29/33	16/55	<0.001
Bilateral PVs flowing into bilateral atriums respectively	4/33	4/55	<0.001
The PVs forming a common lumen at the top of the atrium and flowing back into one side of the atrium	0/33	6/55	0.08
ACPV	0/33	2/55	0.53
Subcardiac TAPVC	0/33	10/55	0.01
Supracardiac TAPVC	0/33	7/55	0.042
Mixed TAPVC	0/33	10/55	0.01
Cardiac malformations			
AVSD	21/33	41/55	0.337
SA	10/33	15/55	0.890
SV	2/33	3/55	>0.9999
TA	0/33	2/55	0.526
LVOTO	7/33	4/55	0.093
AO origining from right ventricle	4/33	4/55	0.467
PS	1/33	26/55	<0.0001
PA	4/33	21/55	0.014
DORV	4/33	7/55	>0.9999
Fetal heart rate rhythm			
Sinus bradycardia	13/33	0/55	<0.0001
Atrioventricular block	4/33	0/55	0.018
Normal	16/33	55/55	<0.0001
Atrial appendage			
Bilateral left atrial appendage	20/33	0/55	<0.0001
Bilateral right atrial appendage	0/33	35/55	<0.0001
Uncertain	3/33	5/55	>0.9999
Unobservable	10/33	15/55	0.810
Bronchus			
Bilateral left bronchus	16/33	0/55	<0.0001
Bilateral right bronchus	0/33	25/55	<0.0001
Uncertain	3/33	5/55	>0.9999
Unobservable	14/33	25/55	0.827
Spleen			
Polysplenia	23/33	1/55	<0.0001
Asplenia	0/33	50/55	<0.0001
Basically normal	10/33	4/55	<0.005

IVC, inferior vena cava; AO, aorta; SVC, superior vena cava; DV, ductus venosus; HV, hepatic vein; PV, pulmonary vein; ACPV, atresia of the common pulmonary vein; AVSD, Atrioventricular septal defects; SA, Single atrium; SV, Single ventricle; TA, Tricuspid atresia; LVOTO, left ventricular outflow tract obstruction; PS, Pulmonary stenosis; PA, Pulmonary atresia; DORV, double-outlet right ventricle.

### Anatomy and casting characteristics of fetal right isomerism

Of the 46 terminated cases, 41 cases received autopsy, which confirmed the prenatal diagnosis, and 5 cases underwent vascular casting. The typical malformations of RI included the right heart, bilateral right atrial appendages (Figure 4D), and APVC (Figures 4E,F). The combined intracardiac abnormalities included AVSD, single atrium, single ventricle, double-outlet right ventricle, and pulmonary arterial stenosis or atresia, etc. The extracardiac malformations included gastric blisters on the right side and the posterior part of the abdominal cavity near the spinal column, the abdominal aorta and IVC arranged on the same side of the spine (Figure 4E), bilateral lungs with three lobes, midline liver (Figure 4F), and asplenia. Atypical signs included one case (2%) of gastric vesicles located in the center of the posterior part of the abdominal cavity and partially herniated into the thoracic cavity, four cases (7%) of a small, thin spleen at the back of the stomach, two cases (4%) of bilateral lungs with four lobes, five cases (9%) of the small left atrial appendage of unclear morphological features, and one case (2%) of double superior vena cava with bridging vein connection.

**Figure 5:**
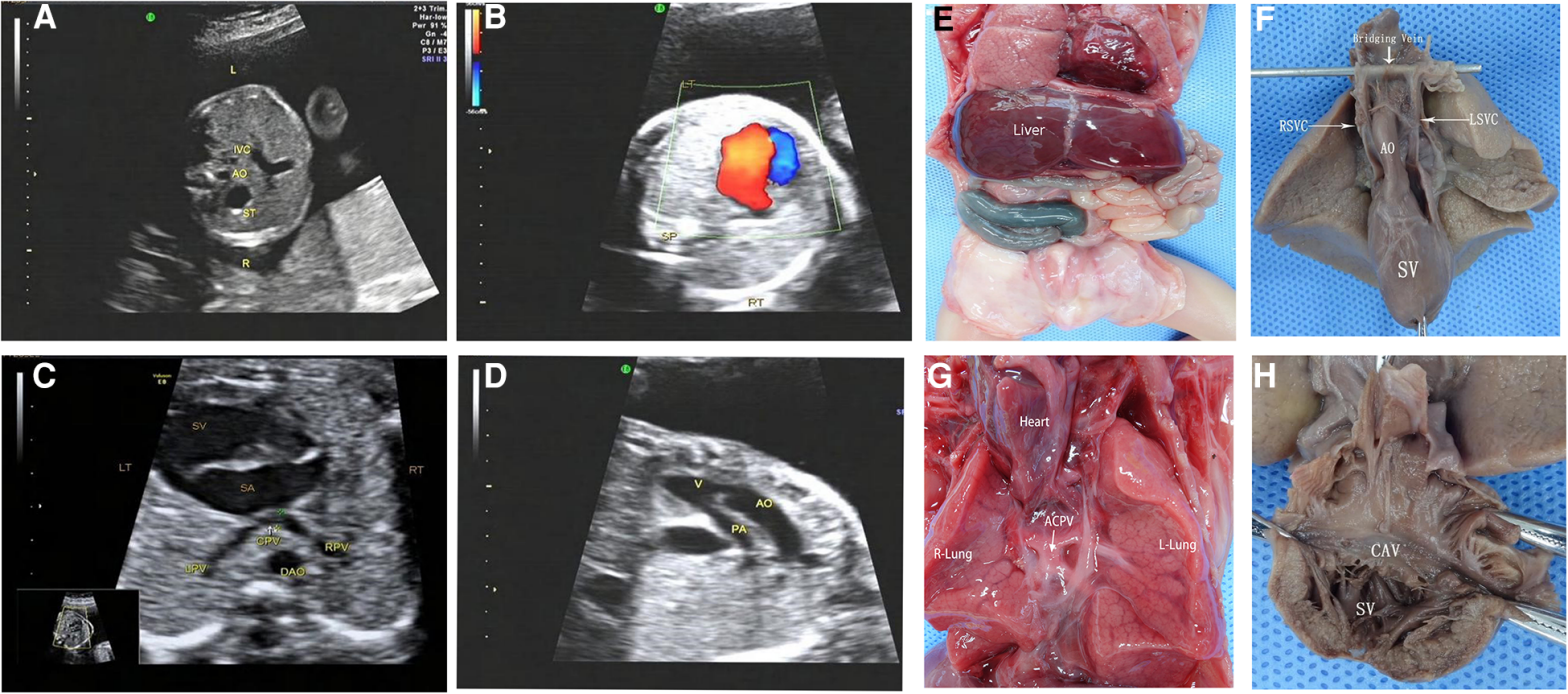
Prenatal ultrasonography and anatomical images of RI fetuses with atresia of common pulmonary vein (ACPV). (**A**) The AO and IVC were on the left side of the spine, and the IVC was in the left front of the abdominal aorta. (**B**) One group of common atrioventricular valve blood flow was seen in the CDFI four-chamber section during the diastolic period. (**C**) Pulmonary veins form a common cavity of the pulmonary vein behind the atrium. (**D**) The aorta and pulmonary artery originated parallelly from the single ventricle, complicated with pulmonary stenosis. Figure (**E–H**) anatomical images. (**E**) Midline liver, intestinal malrotation, the appendix in the right upper abdomen. (**F**) Single ventricle, double superior vena cava with bridging vein connection. (**G**) The heart turned upside down, and the pulmonary veins formed a closed common cavity behind the atrium. (**H**) The heart specimen was clam-opened, exposing a single ventricle consisting mainly of a right ventricle common atrioventricular inlet, and atrioventricular valve. SA, single atrium; SV, single ventricle; LPV, left pulmonary vein; RPV, right pulmonary vein; CPV, common cavity of the pulmonary vein.

One case of the single ventricle was misdiagnosed as an unbalanced atrioventricular septal defect and one case of pulmonary atresia as pulmonary arterial stenosis. In four cases, the spleen was seen in the right posterior part of the stomach, which was misdiagnosed by prenatal ultrasonography. Three cases of APVC were misdiagnosed by ultrasonography, including one case of common vena cava atresia mistaken as complete APVC and two cases as mixed APVC (Figure 5); four APVC cases were overlooked, including one case of abnormal drainage of sub-cardiac pulmonary vein and three cases of common cava atresia of a pulmonary vein; four cases of the hepatic vein were missed by ultrasound scanning.

### Comparison of ultrasound characteristics between LI and RI cases

The fetuses with LI or RI feature typical and shared ultrasound manifestations, especially in the systemic pulmonary venous circulation and cardiac malformations. All malformations of patients with LI and RI were recorded in Table 1. Most HS fetuses (61% of the LI fetuses and 71% of the RI fetuses) showed an inconsistency between the positions of gastric vesicles and the heart. Of note, more than half of the HS cases reported a left-sided heart and right-sided gastric vesicles; only LI cases displayed an absence of a hepatic segment of IVC with AV dilatation (*P* < 0.0001), although the double SVC was found in both LI and RI cases (LI vs. RI: 33% vs. 51%, *P* = 0.11). The reflux characteristics of hepatic veins were observed. Only three LI cases (9%) had hepatic veins and venous ducts flowing into both atria separately, while 76% of RI cases reported hepatic veins flowing into both atria or one atrium separately. Compared with LI, RI showed more abnormal reflux of the hepatic vein and reported more cases of APVC (*P* < 0.05). The intracardiac malformations in LI included AVSD, single atrium, and left ventricular outflow tract obstruction, while the proportion of RI with intracardiac malformations was much higher, including pulmonary stenosis, pulmonary atresia (*P* < 0.05), and double-outlet right ventricle. Fifty-two percent of LI fetuses reported heart conduction problems, such as bradycardia and atrioventricular block, which were not found in the RI cases.

## Discussion

The available literature has documented typical ultrasonic features in LI and RI during an ultrasound examination (2, 13). In our study, after routine ultrasound screening, when the HS cases were preliminarily suspected according to these associative features, they were further subjected to distinction and classification by the proposed five-step prenatal ultrasonic examination, with each individual system of atypical HS fetuses described systemically. The proposed five-step procedure emphasizes the step-by-step recording of the abnormal arrangement of internal organs and extracardiac abnormalities (ECA), which facilitates the depiction of the anomaly spectrum of fetal HS and accurate ultrasound diagnosis of the fetus during early pregnancy. The incidence of anomalies in HS fetuses was consistent with the previous literature (23). In this study, the inconsistency of gastric vesicles with the heart position was reported in 60% of LI and 71% of RI fetuses, which indicates a great possibility of HS. The LI fetuses mostly showed an absent hepatic segment of IVC accompanied by a dilated AV, which is consistent with previous reports (24). Two LI cases did not indicate an absence of the hepatic segment of the IVC but showed a small hepatic segment of the IVC accompanied by the dilatation of the AV. The common cardiac abnormalities of LI are atrioventricular septal defect and single atrium. In LI fetuses, the development or disappearance of the sinus node can lead to sinus bradycardia, borderline escape, and atrioventricular block. 13 cases of bradycardia were reported in the LI group in our current cohort. As the heart block may present in LI fetuses in early pregnancy (25), detecting the heart block in early pregnancy might be crucial for the early prenatal diagnosis of LI.

In the current study, most of the RI fetuses reported APVC. Those who mixed APVC had multiple drainage sites, such as the atrium, portal vein, superior vena cava, and venous catheter. An accurate exploration of the drainage location can provide supportive information for surgical interventions (26). Previous studies seldom focus on the abnormalities of hepatic veins in HS fetuses (27). In the current study, the anomalies involved more than three hepatic veins: which differs from previous reports (28). In 76% of RI cases, hepatic veins and venous ducts flowed into both atriums and both sides of the single atrium, respectively. The potential explanation for this disparity may be the updated examination methods. Right isomerism is often associated with intracardiac malformations, including atrioventricular septal defect, double-outlet right ventricle, pulmonary arterial stenosis or atresia. The five-step prenatal ultrasound examination yields satisfactory reliability. One case was misdiagnosed as a choledochal cyst with no obvious intracardiac malformation, which was confirmed as biliary atresia splenic malformation syndrome (BASM) after birth. The fetus suffered from recurrent cholangitis after the operation and died of liver failure at the age of 1 year. According to the available evidence, biliary atresia may occur in about 10% of LI cases (25). Of the various types of biliary atresia, BASM may develop into cirrhosis and liver failure in the early stage and may be complicated with many other malformations, posing challenges even to liver transplantation and suggesting a poor prognosis (26). Although challenges remain regarding the prenatal ultrasound diagnosis of biliary atresia, efforts should still be made to evaluate the gallbladder-related conditions in the suspected LI fetuses. The possibility of BASM should be indicated in the presence of a small gallbladder that is abnormal in shape and unclear on display or isolated small cysts near the gallbladder.

Atresia of the common pulmonary vein (ACPV) is a rare congenital cardiovascular malformation. The left and right pulmonary veins form a common pulmonary vena cava, which is not connected to the left atrium, right atrium, or other main body veins (29). As ACPV is often located in the dorsal side of the heart, it is prone to missed diagnosis or misdiagnosis as a complete anomalous pulmonary venous connection (30). However, vascular casting greatly assists ultrasonologists in examining intracardiac malformations and systemic and pulmonary venous connections, broadening their understanding of the disease (31). The postnatal mortality rate of ACPV is extremely high, and the afflicted rarely survive for more than 4 weeks (32). The possibility of ACPV should be considered when prenatal ultrasound detects a common pulmonary venous lumen behind the fetal atrium but fails to clearly show the vertical venous drainage of the common pulmonary venous lumen. In such a scenario, the gain of blood flow should be adequately adjusted to prevent excessive gain from being mistaken for the running drainage vessel.

The prognosis of HS is associated with combined intracardiac and extracardiac malformations. When the malformations only involve IVC absence without the complications of other malformations, or complicated with mild cardiac malformations, LI fetuses usually have an acceptable prognosis (4, 33, 34). Therefore, the parents should be encouraged to avoid excessive induction of labor after diagnosis. Fetal edema and IUFD are more likely to occur in LI fetuses with abnormal heart rhythms, especially atrioventricular blocks. In addition, a complete atrioventricular block, in an LI subject, may be associated with myocardial noncompactness, which is characterized by local ventricular wall thickening and deep trabecular fovea. Such association almost always indicates a poor prognosis. RI is usually combined with conotruncal defects, right ventricular outflow tract obstruction and hypoplastic left heart syndrome. Given the high incidence rate of CHD in RI cases, it commonly faces worse perinatal outcomes than LI. In the current cohort, nearly half of the RI fetuses had a complete anomalous pulmonary venous connection, especially in the case of obstruction, which involved high-risk adverse outcomes. The surgical plan may be prescribed for biventricular repair (LI) and single ventricular repair (RI). However, despite these babies having surgery planned, the mortality rate during and after the operation is still high due to the need for staged operations and the difficulties encountered during operation (3). Considering the poor prognosis of HS, most of the family members chose labor termination in this study. However, HS abnormalities should be accurately diagnosed before decision-making. We should provide appropriate counselling and detailed information for the families. When an HS case was suspected after the routine fetal anatomy screening, it underwent rigorous examination and classification following the five-step prenatal ultrasonography.

In general, the gist of the five-step ultrasonic examination is to observe the location of the heart and gastric vesicles, the return of the systemic and pulmonary veins, intracardiac and extracardiac malformations and heart rhythm, especially spleen, liver and other organs. This set of ultrasonic procedures has an important diagnostic value for HS that could be verified by pathological anatomy. HS is caused by various genetic mutations that impact critical developmental pathways during embryogenesis. Characterization of the fetal organs by ultrasound provides diagnostic biomarkers for HS. Further investigation is imperative to provide information on the underlying mechanisms of this rare disease and improve future prenatal management and guidance.

## Limitations

This study is a retrospective analysis of verified diagnoses. Many pregnant women chose termination of pregnancy instead of surgical intervention. We have limited clinical follow-ups. The findings of the patients are only based on the data derived from prenatal ultrasonography.

## Conclusions

The proposed five-step prenatal ultrasonic approach can produce an accurate prenatal diagnosis of HS, which would facilitate timely intervention, prenatal counselling and management. Anatomical casting helps improve the understanding of HS. The different sides of gastric vesicles and cardiac apex are important diagnostic indicators for HS. Disconnection or hypoplasia of IVC, characteristic of complex cardiac malformation, atrioventricular block and BASM are some indicators for left isomerism; For the right isomerism, the diagnostic clues include anomalous pulmonary venous connection, parallel IVC and AO, and intracardiac malformations, such as atrioventricular septal defect, double-outlet right ventricle, and right ventricular outflow tract obstruction.

## Data Availability

The original contributions presented in the study are included in the article, further inquiries can be directed to the corresponding author.
